# Fracture of the Leg

**Published:** 1829-01

**Authors:** 


### FRACTURE OF THE LEG.

### *Fracture of the Leg, communicating with the Ankle-Joint.*

J. West, ætatis forty-one, was admitted into the Worcester Infirmary, September 19, 1825, under the care of Mr. Sheppard, with compound fracture of the leg. On examination, both bones were found fractured about four inches above the ankle, with two small wounds through the skin, corresponding to the fractured ends of the bones. The tibia was also broken immediately above the malleolus internus, and this fracture was discovered to communicate with the ankle-joint. The lower part of the leg and foot was severely bruised. The man's general health was much affected, having been labouring under illness for the last six months.

*Treatment.*—The bones, which were but slightly displaced, by gentle extension were easily brought in apposition with each other; the edges of the wounds were brought together by adhesive plaster, and the leg placed on the side in the semiflexed position. It was bound up in the tail bandage, and a splint placed above and below. The parts to be kept wet with a cold evaporating lotion.

Sept. 20th.—Has had much pain in the leg during the night. Pulse 100; tongue white and dry; skin hot, but moist; there is tenderness on pressure in the epigastric region.

℞. Hydrarg. Subm. gr. v.; Pulv. Jalapæ gr. xv. fiat bol. stat. sum.—Mist. Cath. ℥ij. post horas quatuor.

21st.—Leg rather easier this morning; he had about two hours' sleep during the night. Pulse 100, not hard; tongue no cleaner; bowels rather confined. The pain and tenderness of the epigastric region removed.

Rept^r Haustus Cath.

23d.—The leg dressed this morning: wounds have not united; the contused parts over the tibia, to some extent, are of a livid hue, and appear likely to slough. The febrile symptoms somewhat aggravated.

Sumat Haust. Salin. efferves. quartis horis.—Pulv. Ipecac. Co. gr. x. h. s.—A poultice to be applied to the leg.

26th.—The dead parts are separated, and there is a large unhealthy sore surrounding the tibia, about the size of a crown piece, whereby the fractured ends of the bone are exposed: they are in close contact with each other. There is a small slough over the fibula, but it is merely superficial.

28th.—Wound discharges profusely; has no pain; in other respects much the same. Allowed meat and four ounces of port wine daily.

October 1st.—The leg is improved in appearance, and the wound looks cleaner. Granulations are beginning to show themselves. Has no pain; sleeps well at night; appetite good.

4th.—The wound looks exceeding well; granulations of a florid colour. There is a small opening near the ankle, which probably communicates with the joint, as a fluid is constantly escaping from it, having much the appearance of synovia. The leg keeps in a very good position, and is perfectly easy.

6th.—Continues to improve. Only a very small portion of the bone visible, the rest being covered by granulations; discharge lessened. The poultice discontinued, and the wounds dressed with lint and the chalk cerate.

13th.—Wound begins to cicatrise, and no part of the bone is now visible. Bowels, tongue, and pulse natural.

29th.—The wounds are now quite healed; union between the bones appears to have taken place; health very good.

November 30th.—Discharged cured.

This was a very successful case. The leg was only lifted once during the whole cure, silk oilskin being placed between the bandages and the dressings.

